# Associations Between the Dopamine D4 Receptor and DAT1 Dopamine Transporter Genes Polymorphisms and Personality Traits in Addicted Patients

**DOI:** 10.3390/ijerph15102076

**Published:** 2018-09-21

**Authors:** Jolanta Chmielowiec, Krzysztof Chmielowiec, Aleksandra Suchanecka, Grzegorz Trybek, Bożena Mroczek, Iwona Małecka, Anna Grzywacz

**Affiliations:** 1 Department of Hygiene and Epidemiology, Faculty of Medicine and Health Science, University of Zielona Góra, Zyty 28 St., 65-046 Zielona Gora, Poland; chmiele1@o2.pl (J.C.); chmiele@vp.pl (K.C.); 2 Independent Laboratory of Health Promotion of the Pomeranian Medical University in Szczecin, 11 Chlapowskiego St., 70-204 Szczecin, Poland; o.suchanecka@gmail.com; 3 Department of Oral Surgery, Pomeranian Medical University in Szczecin, 72 Powstańców Wlkp. St., 70-111 Szczecin, Poland; g.trybek@gmail.com; 4 Department of Human Sciences in Medicine, Pomeranian Medical University in Szczecin, 11 Chlapowskiego St., 70-204 Szczecin, Poland; b_mroczek@data.pl; 5 Department of Psychiatry, Pomeranian Medical University in Szczecin, 71-460 Szczecin, Poland; i.malecka@wp.pl

**Keywords:** addiction, genetics, personality traits

## Abstract

Many factors are involved in addiction. The dopaminergic system is thought to be the key element in this process. The mesolimbic dopamine system is a crucial element in the reward system. Changes in this system are thought to be leading to substance use disorders and dependence. Therefore, for our study we chose an analysis of two polymorphisms in genes (Variable Number of Tandem Repeats in *DRD4* and *DAT1*) responsible for dopaminergic transmission, which might be implicated in the scores of personality traits measured by the NEO-FFI test. The study group consisted of 600 male volunteers—299 addicted subjects and 301 controls. Both groups were recruited by psychiatrists; in the case group addiction was diagnosed; in the controls a mental illness was excluded. In both groups the same psychometric test and genotyping by the PCR VNTR method were performed. The results were investigated by a multivariate analysis of the main effects ANOVA. In the presented study no *DRD4* main effects were found for any of the analyzed traits but the *DRD4* main effects approximated to the statistical significance for the extraversion scale. However, no *DAT1* main effects were found for any of the analyzed traits but the *DAT1* main effects approximated to the statistical significance for the agreeability scale.These associations open new possibilities for addiction research.

## 1. Introduction

Substance use disorder is a global health problem. In 2015 about a quarter of a billion people used drugs. Of these, around 29.5 million people—or 0.6 percent of the global adult population—were engaged in problematic use and suffered from drug use disorders, including dependence. Opioids were the most harmful drug type and accounted for 70 percent of the negative health impact associated with drug use disorders worldwide, according to the latest World Drug Report, released by the United Nations Office on Drugs and Crime in 2017 [1]. This data relates only to people aged 15–64. Therefore, a large part of the population is not covered. Consequently, we may safely assume that the actual number of people suffering from drug use disorders is much greater. Dependence is a multifactorial disorder including a complex interaction of factors both genetic and environmental. It emerges in individuals prone to this multifactorial base in response to repeated exposure to addictive substance as well as involves behavioral changes [2]. Many factors are involved in addiction. The dopaminergic system is thought to be the key element in this process. The mesolimbic dopamine system is a crucial element in the reward system. Changes in this system are thought to be leading to substance use disorders and dependence [3]. Many researchers analyzed associations between polymorphisms in genes coding dopamine receptors and the transporter, with contradicting results [4,5]. It seems accurate to analyze a genetic predisposition to addiction, i.e., polymorphic variants of chosen genes, but together with other traits of the phenotype—personality traits [6]. Of course, such attempts have already been made—concerned with different genes and different personality traits—in order to define personality traits which could build ‘addictive personality’ [7], but the association between them is still unknown to us.

Personality is a factor influencing behavior, lifestyle, and shows association with maintaining proper functions in a lifetime. The latest personality research focus on the ‘five factor model, the so called Big Five [8,9,10,11], which consists of five traits: Openness, Conscientiousness, Extraversion, Agreeableness and Neuroticism. These traits have their impact on differences among people and are associated with behavior, emotions, motivation and cognition [12]. A revised NEO personality inventory (NEO-FFI) is used broadly for analyzing these personality traits [9].

Dopamine neurotransmission has been suggested to be associated with novelty seeking [13,14]—a trait defining “excitement to novel stimuli”, associated with dependence [15,16] and relapse [17]. Another personality trait, extraversion, has been linked with the dopaminergic system as well. In the twin studies it was revealed that each trait is influenced by genetics in a different manner—ranging from 25 to 61% [18,19].

The dopamine receptor 4 (*DRD4*) gene is associated with both novelty seeking and extraversion in healthy [20] and dependent [21] subjects. But there are studies that do not confirm this association [22]. The *DRD4* gene is located in chromosome 11p.15.5. The most often analyzed polymorphism of this gene is a variable number of tandem repeats (VNTR) located in its third exon. To date 2 to 10 repeat variants of 48 bp have been identified [23], influencing the length of the third intracellular loop of the D4 receptor. Interestingly, polymorphic variants influence gene expression in a different manner [24]. According to the study by Asghari [25] receptors coded with different length variants differ in sensitivity to endogenous dopamine. Another gene, crucial to the dopaminergic system, is dopamine transporter gene *DAT1*. It is located in chromosome 5p.15.3. Its most analyzed polymorphism is VNTR located in the 3′UTR region. 3 to 13 repeats of 40 bp sequence have been identified, but the most often observed ones are variants with 9 and 10 [26]. There are conflicting results regarding the effect of polymorphic variants and levels of the *DAT1* transcription [27,28]. There are reports of an association between *DAT1* VNTR and novelty seeking [29], but also contradicting ones [30]. The dopamine transporter gene was extensively analyzed by our team in the past in the group of alcohol dependent subjects with results showing an association between the *DAT1* gene and alcohol addiction [31,32].

The main aim of the present study was to analyze VNTR polymorphisms in the *DAT1* and *DRD4* genes in the group of subjects with substance use disorders and in controls. Additionally, we incorporated a personality traits analysis into our examination. Hence, the genetic analysis was combined with a personality traits assessment performed by means of Neo Personality Inventory (NEO-FFI).

## 2. Materials and Methods

The study was conducted in the Independent Laboratory of Health Promotion, Pomeranian Medical University in Szczecin, after obtaining the approval of the Bioethics Committee of the Pomeranian Medical University (KB-0012/106/16) as well as an informed, written consent of the subjects. The study group comprised of 600 male volunteers: drug addicted patients (*n* = 299; mean age = 28, SD = 6.45) and healthy controls (*n* = 301; mean age = 22, SD = 4.57). The addicted subjects were recruited in addiction treatment facilities in the province of Lubuskie after at least 3 months of abstinence. The control group comprised of healthy, non-addicted subjects. Both groups were tested by the psychiatrist; Mini–Mental State Examination (MINI) and the NEO Five-Factor Inventory (NEO-FFI) questionnaires were administered. The history of drug addiction was obtained using the Polish version of ICD 10, medical history and the authors’ survey. DNA was provided from the whole blood aspirated from the elbow vein.

### 2.1. Genotyping

The genomic DNA was isolated from venous blood according to standard procedures. Samples were genotyped using the PCR method. Two polymorphisms were analyzed: *DAT (SLC6A3)* 3′UTR and *DRD4* exon III VNTRs. The *DAT1* genotypes were grouped according to the presence of the 9 and 10 repeat variants. Genotyping was performed by the PCR-VNTR method, using primers: F: 5′-TGT GGT GTA GGG AAC GGC CTG Ag 3′, R: 5′-CTT CCT GGA GGT CAC GGC TCA AGG 3′; in the final volume of 25 µL PCR mix per reaction, with l00 ng genomic DNA, 10 pmol of primers, 50 mM KCL, 10 mM TrisHCl, 1.5 mM MgCl2, 200 µM dATP, dCTP, dTTP, dGTP and 0.8 U of the Tag polymerase. Conditions for reaction: 5 min. of initial denaturation in 94 °C, cycling—55 s. of denaturation in 94 °C, 50 s. of primers hybridization in 55 °C and 1 min. of elongation in 72 °C, repeated in 30 cycles, 10 min. of final elongation in 72 °C. The amplified products were visualized using ethidium bromide stained gel electrophoresis (3% agarose) and UV photography. The products lengths were 450 bp for 10 repeats allele and 410 bp for 9 repeats allele.

The *DRD4* genotypes were grouped based on the presence of the short (2–5 repeat) and long (6–11 repeat) variants. Genotyping was performed by the PCR-VNTR method, using primers: F: 5′-GCG ACT ACG TGG TCT ACT CG 3′, R: 5′-AGG ACC CTC ATG GCC TTG 3′; in the final volume of 25 µL PCR mix per reaction, with l00 ng genomic DNA, 10 pmol of primers, 50 mM KCL, 10 mM TrisHCl, 1.5 mM MgCl2, 200 µM dATP, dCTP, dTTP, dGTP and 0.8 U of the Tag polymerase. Conditions for reaction: 3 min. of initial denaturation in 95 °C, cycling—30 s. of denaturation in 95 °C, 1 min. of primers hybridization in 63 °C and 30 s. of elongation in 72 °C, repeated in 35 cycles, 5 min. of final elongation in 72 °C. The amplified products were visualized using ethidium bromide stained gel electrophoresis (3% agarose) and UV photography. The products ranged from 379 bp (2 repeats) to 811 (11 repeats). The products were divided into 2 groups: short alleles (2–5 repeats) and long alleles (6–11 repeats).

### 2.2. Statistical Analysis

Analysis of differences between healthy controls and addicted subjects in NEO Five Factor Inventory results was performed using the Student’s *t*-test (t). The frequencies of genotypes and alleles of the *DAT1* and *DRD4* genes polymorphisms in patients with drug addiction and in controls were studied using the chi square test (χ^2^).

The relations between the genotype variant and NEO Five Factor Inventory (NEO-FFI, Mean M and standard deviation SD) were studied by means of a multivariate analysis of main effects ANOVA (genetic feature × control and addicted subjects). The Bonferroni multiple comparisons correction was applied. The accepted level of significance was 0.01 (0.05/5). All computations were performed using STATISTICA 13 (Tibco Software Inc, Palo Alto, CA, USA) for Windows (Microsoft Corporation, Redmond, WA, USA).

## 3. Results

In comparison with the controls the case group subjects had significantly higher scores on the scale of Neuroticism (M 6.73 vs. M 4.67, *p* ≤ 0.01), Openness (M 5.01 vs. M 4.53, *p* ≤ 0.01), and lower scores on the scales of Extraversion (M 5.76 vs. M 6.37, *p* ≤ 0.01), Agreeability (M 4.30 vs. M 5.59, *p* ≤ 0.01) and Conscientiousness (M 5.59 vs. M 6.08, *p* ≤ 0.01) Table 1.

In comparison with the control group a statistically significant difference in the genotype frequency for the *DRD4* gene in addicted subjects was found (s/s 0.63 vs. s/s 0.59, s/l 0.33 vs. s/l 0.33, l/l 0.03 vs. l/l 0.08, χ^2^ = 7.62, *p* = 0.022) as well as a statistically significant difference in the frequency for the *D4D4* between the addicted subjects and the control group (s 0.8 vs. s 0.75, l 0.2 vs. l 0.25, χ^2^ = 4.05, *p* = 0.044). However, no statistically significant difference was found between the addicted subjects and the control group in the frequency for the *DAT1* genotypes (10/10 0.56 vs. 10/10 0.56, 9/10 0.41 vs. 9/10 0.38, 9/9 0.03 vs. 9/9 0.06, χ^2^ = 3.92, *p* = 0.141) and the frequency of *DAT1* alleles (10 0.77 vs. 10 0.75, 9 0.23 vs. 9 0.25, χ^2^ = 0.55, *p* = 0.460) Table 2.

### 3.1. DRD4 Variant Interaction

The full model ANOVA of addicted subjects and control subjects and the *DRD4* variant interaction was found for the Neuroticism scale (F**_3,595_** = 48.75, *p* ≤ 0.01, R^2^ = 0.197), Extraversion scale (F**_3,595_** = 6.31, *p* ≤ 0.01, R^2^ = 0.031), Openness scale (F_3,595_ = 4.87, *p* ≤ 0.01, R^2^ = 0.024), Agreeability scale (F**_3,595_** = 21.95, *p* ≤ 0.01, R^2^ = 0.100).

The main effect of addicted subjects and control subjects was found for the Neuroticism scale (F_1,595_ = 139.30, *p* ≤ 0.01, η^2^ = 0.19, observed power = 1.00), Extraversion scale (F_1,595_ = 11.73, *p* ≤ 0.01, η^2^ = 0.019, observed power = 0.93), Openness scale (F_1,595_ = 11.14, *p* ≤ 0.01, η^2^ = 0.018, observed power = 0.91), and Agreeability scale (F_1,595_ = 64.36, *p* ≤ 0.01, η**^2^** = 0.098, observed power = 1.00). No *DRD4* main effects were found for any of the analyzed traits but the *DRD4* main effects approximated to the statistical significance for the Extraversion scale (F_2,595_ = 2.76, *p* = 0.064, η^2^ = 0.01, observed power = 0.54). The means and standard deviations for all NEO Five Factor Inventory for the *DRD4* variant interaction addicted subjects and control subjects are presented in Table 3.

### 3.2. DAT1 Variant Interaction

The full model ANOVA of addicted subjects and control subjects and *DAT1* variant interaction was found for the Neuroticism scale (F_3,595_ = 48.16, *p* ≤ 0.01, R^2^ = 0.195), Extraversion scale (F_3,595_ = 4.88, *p* ≤ 0.01, R^2^ = 0.024), Agreeability scale (F_3,595_ = 22.87, *p* ≤ 0.01, R^2^ = 0.103).

The main effect of addicted subjects and control subjects was found for the Neuroticism scale (F_1,595_ = 143.28, *p* ≤ 0.01, η^2^ = 0.194, observed power = 1.00), Extraversion scale (F_1,595_ = 13.51, *p* ≤ 0.01, η^2^ = 0.022, observed power = 0.96), Openness scale (F_1,595_ = 9.97, *p* ≤ 0.01, η^2^ = 0.016, observed power = 0.88), Agreeability scale (F_1,595_ = 64.55, *p* ≤ 0.01, η^2^ = 0.098, observed power = 1.00) and Conscientiousness scale (F_1,595_ = 6.89, *p* ≤ 0.01, η^2^ = 0.01, observed power = 0.74). No *DAT1* main effects were found for any of the analyzed traits, but *DAT1* main effects approximated to the statistical significance for the Agreeability scale (F_2,595_ = 2.81, *p* = 0.061, η^2^ = 0.01, observed power = 0.55). The means and standard deviations for all NEO Five Factor Inventory for the *DAT1* variant interaction addicted subjects and control subjects are presented in Table 4.

In the analysis of ANOVA variants, both for *DRD4* and *DAT1*, no significant influence on interactions between the genetic factor and addiction or its absence (addicted/control x *DRD4* Ex3, addicted/control x *DAT1*) was found to have any connection with the NEO-FFI results.

Finally, based on our power calculation, our sample had more than 0.74 observed power to detect addicted subjects and control subjects’ main effects of the studied NEO Five Factor Inventory and their interaction effect (19% to approximately 2% of the phenotype variance found explanation).

## 4. Discussion

In our study we focused on combining personality traits measured by the NEO-FFI test and genetic factors in the context of aspects related to the occurrence of addiction.

Genetics of personality has been studied for many years now. Of course, single SNPs do not condition personality traits but analysis, such as ours, but combining genetic and personality factors in addicted subjects seems to be a proper way for deciphering what impact those factors have on addiction [33]. Especially, when analyzing the two most important genes—*DRD4* and *DAT1* of the reward system.

In our study we discovered an increased frequency of *DRD4* s/s genotype and s allele in the control group, which is very uncommon, because in most studies there is an association either with long alleles [34,35], or there is none [36,37]. As for analysis of *DAT1* genotypes and allele frequencies we did not find any differences in distribution among our subjects. These results are in line with the previous study by Oniszczenko and Dragan [6].

In our study no *DRD4* main effects were found for any of the analyzed traits, but the *DRD4* main effects approximated to the statistical significance for the extraversion scale, which confirms studies associating the *DRD4* gene with extraversion [38]. In the light of literature reports discussed below, the polymorphism of the *DRD4* gene seems to be particularly interesting. It is associated with novelty seeking [39,40,41], and this trait is in turn connected with predisposition to addiction [42,43,44]. In 2001 a team of researchers found an association between the *DRD4* gene and novelty seeking in addicted subjects. However, researchers concluded that the polymorphic variant of this gene is not a factor conditioning addiction but might predispose people who abuse substances to develop more extreme forms of addiction [45].

The interaction between novelty seeking and alcohol consumption has been analyzed by Bau and colleagues [46]. The two polymorphisms of *DRD4* and *DAT* analyzed in our study were also analyzed in the group of 587 patients, but the study did not find any significant associations. The study performed on the *DRD4* knockout mice suggest an important role of the analyzed gene in exploratory behavior and increased alcohol consumption. Interestingly, these correlations were only present in male subjects [47]. Although, not all knockout mice studies confirms this finding [48]. The genetic aspects of addiction have been studied by many researchers. One of them is Erjavec and colleagues [49] who conducted a research on a substantial group of alcohol addicted patients (*n* = 690) vs. controls (*n* = 580) concerning the *MAOB* and *DRD4* genes, considering different phenotypic subtypes, i.e., withdrawal symptoms, aggressive behavior, severity of alcohol dependence, delirium tremens, comorbid depression, suicidal behavior, lifetime suicide attempt and early/late onset of alcohol abuse. Interestingly, associations were present in homogenous subgroups.

Other research concerning the *DRD4* gene was performed by Soyka and colleagues [50]. For their analysis they chose the above gene and personality traits measured by TCI and NEO-FFI. And yet again, no associations were found.

In our study no *DAT1* main effects were found for any of the analyzed traits but the *DAT1* main effects approximated to the statistical significance for the agreeability scale and conscientiousness scale.

The analysis of the *DAT1* gene and personality traits measured by the TPQ test was also performed by Tzeng and colleagues [51] in a large cohort (568 patients vs. 341 controls) of amphetamine addicted Han Chinese male subjects. The researchers found a weak association between the rs27072 polymorphism and development of amphetamine addiction. This finding was neither confirmed by haplotype analysis nor logistic regression. Interestingly, novelty seeking, and harm avoidance scores were higher in the case group than in controls, but the analyzed polymorphism did not influence these scores. Of course, both harm avoidance and novelty seeking are risk factors for developing amphetamine addiction, but the *DAT1* gene does not impact the susceptibility to addiction in the Han Chinese population.

In 2015 researchers went even further—Genome Wide Association Study (GWAS) for personality traits was performed, because the GWA analysis is the best way to show biological pathways in the context of addiction. The study [52] was conducted on a group of 1089 Korean women. Personality traits scores were obtained by NEO-FFI. Genetic analysis included an analysis of 1042 pathways containing 8297 genes. In this study there were no associations between genes analyzed in our study and personality traits scores. We need to bear in mind the fact that both populations differ in terms of sex of the subjects and their ethnicity, which may have its impact on the results.

Another study shows that the impulsivity trait might be heritable risk factors and probable endophenotype of addiction and other mental illnesses including disinhibition. Researchers analyzed the genetic basics of impulsivity, measured by scores on the Barrat’s Impulsivity Scale (BIS-11) and Impulsive Behaviour Scale (UPPS-P) in a group of 983 heathy young adults. There were no significant associations for *DAT1* or *DRD4* genes [53].

Our study in not free from some limitations. We decided to include only male subjects in our study. This decision was based on an extensive body of literature covering the subject of sex differences in psychoactive substances use and abuse, use patterns, phases of developing addiction and side effects. The differences between sexes are present in both human and rodents [54,55]. Hence, we strongly feel that our study need replication in a female study group and in different populations since all our subjects are of Caucasian origin.

We believe that one day our research may contribute to developing public health applications helping those in need for early screening. However, for now, given the early stages of personality x genetics x addiction research and level of significance in our study, we consider it purely a basic research.

So far there has been no success in revealing clear associations combining addictions, personality traits and genetics. The studies tackled this task using different psychometric tests and dividing subjects into homogenous subgroups. This confirms the validity of such analyses and provides the basis for further searches in this area. In our study the analysis revealed that the main effects of *DRD4* gene approximated to the statistical significance for the extraversion scale, and the *DAT1* main effects approximated to the statistical significance for the agreeability scale. Therefore, it seems reasonable to enlarge the study group and to test a greater number of gene polymorphisms associated with personality traits.

## 5. Conclusions

In the presented study no main effects of *DRD4* were found for any of the analyzed traits but the *DRD4* main effects approximated to the statistical significance for the extraversion scale. However, no *DAT1* main effects were found for any of the analyzed traits but the *DAT1* main effects approximated to the statistical significance for the agreeability scale. These associations open new possibilities for addiction research.

## Figures and Tables

**Table 1:** NEO Five Factor Inventory results between healthy control and addicted subjects.

NEO Five Factor Inventory	Addicted Subjects (n = 299)	Control (n = 301)	t df 2	*p* Value
Neuroticism/scale	M 6.73 SD 2.18	M 4.67 SD 2.01	**12.03**	**0.0000**
Extraversion/scale	M 5.76 SD 2.14	M 6.37 SD 1.98	**−3.65**	**0.0003**
Openness/scale	M 5.01 SD 2.02	M 4.53 SD 1.61	**3.206**	**0.0014**
Agreeability/scale	M 4.30 SD 1.93	M 5.59 SD 2.09	**−7.91**	**0.0000**
Conscientiousness/scale	M 5.59 SD 2.27	M 6.08 SD 2.15	**−2.72**	**0.0066**

Bonferroni correction was used, and the *p* value was reduced to 0.01 (*p* = 0.05/5 (number of statistical tests conducted)). M—mean, SD—standard deviation, *t*-Student’s test Significances between-group differences are marked in bold.

**Table 2:** Frequency of genotypes and alleles of the *DAT1* and *DRD4* genes polymorphisms in patients with drug addiction and in controls.

Group	*DRD4* Genotypes Alleles					*DAT1* Genotypes Alleles				
	s/s n (%)	s/l n (%)	l/l n (%)	s n (%)	l n (%)	10/10 n (%)	9/10 n (%)	9/9 n (%)	10 n (%)	9 n (%)
Addicted subjects *n* = 299	189 (0.63)	100 (0.33)	10 (0.03)	478 (0.8)	120 (0.2)	168 (0.56)	122 (0.41)	9 (0.03)	458 (0.77)	140 (0.23)
Control *n* = 301	177 (0.59)	98 (0.33)	26 (0.08)	452 (0.75)	150 (0.25)	168 (0.56)	114 (0.38)	19 (0.06)	450 (0.75)	152 (0.25)
χ^2^ (df) *p* value	**7.62 (2)** **0.022**			**4.05 (1)** **0.044**		3.92 (2) 0.141			0.55 (1) 0.460	

*p*-statistical significance χ^2^ test, *n*—number of subjects. Significances between-group differences are marked in bold.

**Table 3:** Differences in *DRD4* and NEO Five Factor Inventory between healthy control subjects and addicted subjects.

NEO Five Factor Inventory			*DRD4* Ex3			Main Effects ANOVA			
	Addicted Subjects (n = 299)	Control (n = 301)	s/s (n = 365)	s/l (n = 197)	l/l (n = 36)	Full Model	Main Effects		
						F (*p* Value)	Factor	F (*p* Value)	η^2^
Neuroticism/scale	M 6.73 SD 2.18	M 4.67 SD 2.01	M 5.77 SD 2.27	M 5.71 SD 2.27	M 4.81 SD 2.01	**F_3,595_ = 48.75** ***p* = 0.000000** **R^2^ = 0.197**	intercept	**F_1,595_ = 1768.22 (*p* = 0.000000)**	**0.748**
							addicted/control	**F_1,595_ = 139.30 (*p* = 0.000000)**	**0.190**
							*DRD4* Ex3	F_2,595_ = 0.84 (*p* = 0.433326)	0.003
Extraversion/scale	M 5.76 SD 2.14	M 6.37 SD 1.98	M 6.08 SD 2.03	M 5.89 SD 2.15	M 6.89 SD 2.07	**F_3,595_ = 6.31** ***p* = 0.000321** **R^2^ = 0.031**	intercept	**F_1,595_ = 2328.11 (*p* = 0.000000)**	**0.796**
							addicted/control	**F_1,595_ = 11.73 (*p* = 0.000657)**	**0.019**
							*DRD4* Ex3	F_2,595_ = 2.76 (*p* = 0.063836)	0.009
Openness/scale	M 5.01 SD 2.02	M 4.53 SD 1.61	M 4.66 SD 1.81	M 4.94 SD 1.88	M 4.97 SD 1.90	**F_3,595_ = 4.87** ***p* = 0.002353** **R^2^ = 0.024**	intercept	**F_1,595_ = 1804.89 (*p* = 0.000000)**	**0.752**
							addicted/control	**F_1,595_ = 11.14 (*p* = 0.000897)**	**0.018**
							*DRD4* Ex3	F_2,595_ = 2.15 (*p* = 0.117517)	0.007
Agreeability/scale	M 4.30 SD 1.93	M 5.59 SD 2.09	M 4.90 SD 2.16	M 5.09 SD 2.01	M 4.75 SD 2.21	**F_3,595_ = 21.95** ***p* = 0.000000** **R^2^ = 0.100**	intercept	**F_1,595_ = 1446.57 (*p* = 0.000000)**	**0.709**
							addicted/control	**F_1,595_ = 64.36 (*p* = 0.000000)**	**0.098**
							*DRD4* Ex3	F_2,595_ = 1.57 (*p* = 0.209316)	0.005
Conscientiousness/scale	M 5.59 SD 2.27	M 6.08 SD 2.15	M 5.80 SD 2.25	M 5.79 SD 2.19	M 6.44 SD 2.03	F_3,595_ = 3.14 *p* = 0.024785 R^2^ = 0.016	intercept	**F_1,595_ = 1832.62 (*p* = 0.000000)**	**0.755**
							addicted/control	F_1,595_ = 6.52 (*p* = 0.010937)	0.011
							*DRD4* Ex3	F_2,595_ = 1.004 (*p* = 0.366928)	0.003

Bonferroni correction was used, and the *p* value was reduced to 0.01 (*p* = 0.05/5 (number of statistical tests conducted)). M—mean, SD—standard deviation. Significances between-group differences are marked in bold.

**Table 4:** Differences in *DAT1* and NEO Five Factor Inventory between healthy control subjects and addicted subjects.

NEO Five Factor Inventory			*DAT1*			Main Effects ANOVA			
	Addicted Subjects (n = 299)	Control (n = 301)	10/10 (n = 336)	9/10 (n = 234)	9/9 (n = 28)	Full model	Main Effects		
						F (*p* Value)	Factor	F (*p* Value)	η^2^
Neuroticism/scale	M 6.73 SD 2.18	M 4.67 SD 2.01	M 5.66 SD 2,29	M 5.78 SD 2.35	M 5.36 SD 2.74	**F_3,595_ = 48.16** ***p* = 0.000000** **R^2^ = 0.195**	intercept	**F_1,595_ = 1544.33 (*p* = 0.000000)**	**0.722**
							addicted/control	**F_1,595_ = 143.28 (*p* = 0.000000)**	**0.194**
							*DAT1*	F_2,595_ = 0.12 (*p* = 0.885122)	0.0004
Extraversion/scale	M 5.76 SD 2.14	M 6.37 SD 1.98	M 6.15 SD 2.05	M 5.97 SD 2.09	M 5.93 SD 2.32	**F_3,595_ = 4.88** ***p* = 0.002317** **R^2^ = 0.024**	intercept	**F_1,595_ = 1763.17 (*p* = 0.000000)**	**0.747**
							addicted/control	**F_1,595_ = 13.51 (*p* = 0.000259)**	**0.022**
							*DAT* *1*	F_2,595_ = 0.66 (*p* = 0.515363)	0.002
Openness/scale	M 5.01 SD 2.02	M 4.53 SD 1.61	M 4.79 SD 1.84	M 4.77 SD 1.85	M 4.54 SD 1.89	F_3,595_ = 3.49 *p* = 0.015634 R^2^ = 0.017	intercept	**F_1,595_ = 1391.91 (*p* = 0.000000)**	**0.701**
							addicted/control	**F_1,595_ = 9.97 (*p* = 0.001670)**	**0.016**
							*DAT1*	F_2,595_ = 0.11 (*p* = 0.899619)	0.0003
Agreeability/scale	M 4.30 SD 1.93	M 5.60 SD 2.09	M 5.09 SD 2.09	M 4.81 SD 2.14	M 4.50 SD 2.01	**F_3,595_ = 22.87** ***p* = 0.000000** **R^2^ = 0.103**	intercept	**F_1,595_ = 1162.13 (*p* = 0.000000)**	**0.661**
							addicted/control	**F_1,595_ = 64.55 (*p* = 0.000000)**	**0.098**
							*DAT1*	F_2,595_ = 2.81 (*p* = 0.060746)	0.009
Conscientiousness/scale	M 5.59 SD 2.27	M 6.08 SD 2.15	M 5.80 SD 2.26	M 5.80 SD 2.14	M 6.39 SD 2.48	F_3,595_ = 2.92 *p* = 0.03354 R^2^ = 0.014	intercept	**F_1,595_ = 1522.04 (*p* = 0.000000)**	**0.719**
							addicted/control	**F_1,595_ = 6.89 (*p* = 0.008897)**	**0.011**
							*DAT1*	F_2,595_ = 0.671 (*p* = 0.511773)	0.002

Bonferroni correction was used, and the *p* value was reduced to 0.01 (*p* = 0.05/5 (number of statistical tests conducted)). M—mean, SD—standard deviation. Significances between-group differences are marked in bold.
